# Wnt Activity and Cell Proliferation Are Coupled to Extracellular Vesicle Release in Multiple Organoid Models

**DOI:** 10.3389/fcell.2021.670825

**Published:** 2021-06-24

**Authors:** Gyöngyvér Orsolya Sándor, András Áron Soós, Péter Lörincz, Lívia Rojkó, Tünde Harkó, Levente Bogyó, Tamás Tölgyes, Attila Bursics, Edit I. Buzás, Judit Moldvay, Zoltán Wiener

**Affiliations:** ^1^Department of Genetics, Cell and Immunobiology, Semmelweis University, Budapest, Hungary; ^2^Department of Anatomy, Cell and Developmental Biology, Eötvös Loránd University of Sciences, Budapest, Hungary; ^3^Premium Postdoctoral Research Program, Hungarian Academy of Sciences, Budapest, Hungary; ^4^1st Department of Pulmonology, National Korányi Institute of Pulmonology, Budapest, Hungary; ^5^Department of Pathology, National Korányi Institute of Pulmonology, Budapest, Hungary; ^6^Department of Thoracic Surgery, Semmelweis University and National Institute of Oncology, Budapest, Hungary; ^7^Department of Thoracic Surgery, National Korányi Institute of Pulmonology, Budapest, Hungary; ^8^Department of General Surgery and Surgical Oncology, Uzsoki Hospital, Budapest, Hungary; ^9^ELKH-SE Immune-Proteogenomics Extracellular Vesicle Research Group, Semmelweis University, Budapest, Hungary; ^10^HCEMM-SE Extracellular Vesicle Research Group, Budapest, Hungary; ^11^SE-NAP Brain Metastasis Research Group, 2nd Department of Pathology, Semmelweis University, Budapest, Hungary

**Keywords:** lung adenocarcinoma, lung cancer, organoid, pancreatic ductal adenocarcinoma, exosome, CD133, prominin-1

## Abstract

Extracellular vesicles (EV) are considered as a potential tool for early disease diagnosis; however, factors modifying EV release remain partially unknown. By using patient-derived organoids that capture the cellular heterogeneity of epithelial tissues, here we studied the connection between the Wnt-producing microniche and EV secretion in multiple tissues. Although nearly all cells in pancreatic ductal (PD) and pancreatic ductal adenocarcinoma (PDAC) samples expressed porcupine (PORCN), an enzyme critical for Wnt secretion, only a subpopulation of lung bronchiolar (NL) and lung adenocarcinoma (LUAD) organoid cells produced active Wnt. The microniche for proliferating cells was shaped not only by PORCN + cells in NL and LUAD organoids but also by fibroblast-derived EVs. This effect could be blocked by using Wnt secretion inhibitors. Whereas inhibiting Wnt secretion in PD NL or LUAD organoids critically changed both cell proliferation and EV release, these were uncoupled from each other in PDAC. Sorting for CD133 identified a cell population in the LUAD microniche that produced organoids with a high percentage of PORCN + and proliferating cells and an elevated EV secretion, which may explain that CD133 marks LUAD cells with malignant behavior. Collectively, we show here that high cell proliferation rate, induced by Wnt pathway activation, is coupled to a higher EV release, a critical finding that may be considered when developing EV-based diagnostic tools.

## Introduction

Extracellular vesicles (EV) are membrane-surrounded structures released by virtually all cell types. They carry biologically active molecules, such as RNAs, lipids, and proteins from the releasing to the target cells, thus providing a novel way of intercellular communication (Mathieu et al., 2019). EVs transport their cargo in a protected form. Furthermore, they contain molecules specific for the releasing cells at a high concentration in the body fluids and tissues. Thus, they provide a promising tool for early disease diagnosis. In addition, EVs can be edited and loaded with specific molecules, and they may be used in cancer therapies as well (Buzas et al., 2014). The application of EVs as diagnostic tools is critically influenced not only by the molecular cargo but also by the amount of EVs derived from different cell subpopulations in the tissues and body fluids. However, general mechanisms and factors modifying EV release remain poorly known.

Organoids are derived from animal or patient samples, cultured in 3D matrices, such as Matrigel, under well-defined conditions, and maintain the cellular heterogeneity characteristic for the *in vivo* epithelial tissues (Drost and Clevers, 2018). Thus, they represent one of the most modern technologies to study human diseases and enable investigation of mechanisms and factors affecting EV release. Here we used the organoid technology to study different types of normal tissues and cancers.

Colorectal cancer (CRC) is one of the most frequent cancers. In more than 80% of CRC cases, mutation in the *APC* gene is an initializing genetic change, leading to the continuous and ligand-independent activation of the Wnt pathway (Kinzler and Vogelstein, 1996). By using the organoid technology, we have shown previously that *APC* mutation and Wnt activation led to a markedly elevated EV release from intestinal epithelial cells (Szvicsek et al., 2019).

Lung cancer is one of the leading causes of cancer-related death worldwide. Non-small cell lung cancer (NSCLC) accounts for more than 85% of lung cancer cases, and it forms a heterogenous and aggressive disease with a low 5-year survival. The most frequent subtype is lung adenocarcinoma (LUAD), which is often modeled in mice carrying oncogenic *KRas* and inactivating *Trp53* mutations (Muzumdar et al., 2016). Importantly, LUAD patients show a large variety of genetic changes, and only 25 and 50% of patients carry *KRAS* and *TP53* mutations, respectively (Cancer Genome Atlas Research Network, 2014). Thus, although mouse models have provided important insights into LUAD tumorigenesis, this, however, highlights the need for studies using organoid models that capture the mutational heterogeneity of human tumors. Interestingly, although components of the Wnt signaling pathway are rarely mutant in LUAD, a recent study reported the presence of Wnt-producing and Wnt-responding cells in mouse models of this disease. These cells were found to create intratumoral functional heterogeneity via microenvironmental niches with different Wnt activities. Furthermore, it was shown that the Wnt-producing niche drives the proliferative potential and progression in LUAD (Tammela et al., 2017). The secretion of active Wnt proteins requires their palmitoylation by the enzyme porcupine (PORCN) (Barrott et al., 2011), and cells of this Wnt-producing niche express Porcn (Tammela et al., 2017). Importantly, a recently established organoid library highlighted the importance of Wnt signaling at the initial stages of yet another type of tumor, the pancreatic ductal adenocarcinoma (PDAC) (Seino et al., 2018) that has an extremely low 5-year survival with less than 8%. Thus, these results highlight the central role of the Wnt pathway in establishing the cellular heterogeneity not only in CRC and the intestine but also in other cancer types as well. However, whether the inducing effect of Wnt signaling and cell proliferation on EV release is a general hallmark of many normal tissues and tumors is not yet known.

By using pancreatic ductal, PDAC, lung bronchiolar, and LUAD organoids, here we provide evidence that Wnt activity and cell proliferation are coupled to EV release in multiple normal tissue types, but not in all cancers. We show the presence of Wnt-producing cells in lung and LUAD, creating a special microenvironment for proliferating cells in the organoids. This cellular heterogeneity is critically modified by fibroblast-derived EVs transmitting Wnt activity. Furthermore, organoids derived from CD133^*high*^ LUAD cells that represent an aggressive tumor cell population contain a higher number of Wnt-producing and proliferating cells and release more EVs compared to CD133^*low*/–^ cells.

## Materials and Methods

### Cell Culture

H1975 (ATCC CRL-5908, American Type Culture Collection, Manassas, VA, United States) lung cancer cells were cultured in RPMI 1640 Medium (Gibco, Life Technologies, Carlsbad, CA, United States), 10% fetal bovine serum (FBS) (Biosera, Kansas, MO, United States), glutamine (Merck, Darmstadt, Germany), 1X penicillin/streptomycin (Gibco, Waltham, MA, United States), and cyprofloxacine (Merck, Darmstadt, Germany, 1:200 dilution). A549 (ATCC CRL-185) lung cancer cells were cultured in Dulbecco’s Modified Eagle Medium (DMEM) containing 4,500 g/l glucose (Gibco, Thermo Fisher, Waltham, MA, United States), 10% FBS (Biosera, Kansas, MO, United States), glutamine (Merck, Darmstadt, Germany), 1X penicillin/streptomycin (Gibco, Waltham, MA, United States), and cyprofloxacine (Merck, Darmstadt, Germany, 1:200 dilution). BEAS-2B immortalized control bronchiolar cells (ATCC CRL-9609) were maintained in BEBM Bronchial Epithelial Cell Growth Basal Medium (Lonza, Basel, Switzerland, CC-3171) and BEGM Bronchial Epithelial Cell Growth Medium SingleQuots Supplements and Growth Factors (CC-4175, Lonza, Basel, Switzerland), but without adding gentamycin–amphotericin B mix according to ATCC’s recommendations. In some experiments, cells were trypsinized with TrypLE (Gibco, Waltham, MA, United States), washed with phosphate buffered saline (PBS) three times, and embedded into growth factor-reduced, phenol red-free Matrigel (Corning, Corning, NY, United States) at 20,000 cells/25 μl in 48-well plates (Eppendorf, Hamburg, Germany) or on eight-well chamber slides (Corning, New York, NY, United States) to obtain spheroids. Cell number was counted in a Burker chamber. Two days before starting EV collection, the medium was changed to serum-free medium and EVs were harvested after 48 h. We used cells only with low (< p10) passage numbers after receiving them from ATCC. Cell cultures were tested for *Mycoplasma* contamination with Hoechst staining, and they were negative in our studies.

### Mouse Pancreas Ductal and Lung Organoid Cultures

The Pest County Government Office of Hungary (the competent veterinary authority) approved the experiments and the maintainance of mice. Animals were housed in IVC racks, on a cycle of 12L/12D, and experiments were carried out with the approval of the Semmelweis University Animal Care and Use Committee. After the pancreata from C57Bl/6J mice (000664, The Jackson Laboratory, Bar Harbor, ME, United States) were digested with collagenase XI (Sigma, Saint Louis, MO, United States), dispase II (Gibco, Waltham, MA, United States), and DNase I (Merck, Darmstadt, Germany) in DMEM/F12 for 1 h at 37°C, pancreatic ducts isolated under microscope were embedded in Matrigel (Huch et al., 2013). They were then cultured in DMEM/F12 supplemented with 2% antibiotic/antimycotic mix and B27 supplement (Gibco, Waltham, MA, United States), gastrin (10 nM, Merck, Darmstadt, Germany), 1.25 mM *N*-acetyl-cysteine (Sigma, Saint Louis, MO, United States), HEPES buffer (10 mM, Sigma, Saint Louis, MO, United States), mouse R-Spondin 1 (500 ng/ml, R&D Systems, BioTechne, Minneapolis, MN, United States), murine noggin (100 ng/ml, PeproTech, Rocky Hill, NJ, United States), FGF-10 (100 ng/ml, PeproTech, Rocky Hill, NJ, United States), EGF (50 ng/ml, PeproTech, Rocky Hill, NJ, United States), and nicotinamide (10 mM, Merck, Darmstadt, Germany). For lung organoids, tissues isolated from mice were digested with collagenase XI (Sigma, Saint Louis, MO, United States) in DMEM/F12 for 1 h at 37°C. After filtering the cell suspension through a 70-μm cell strainer (Fisherbrand, Fisher Scientific, Waltham, MA, United States), the cells embedded in Matrigel were cultured in DMEM/F12 supplemented with 2% antibiotic/antimycotic mix and B27 supplement (Gibco, Waltham, MA, United States), 1.25 mM *N*-acetyl-cysteine (Sigma, Saint Louis, MO, United States), 10 mM HEPES buffer (Sigma, Saint Louis, MO, United States), mouse R-Spondin 1 (500 ng/ml, R&D Systems, BioTechne, Minneapolis, MN, United States), murine noggin (100 ng/ml, PeproTech, Rocky Hill, NJ, United States), FGF-10 (100 ng/ml, PeproTech, Rocky Hill, NJ, United States), ROCK inhibitor (Y-27632, 5 μM, MedChemExpress, Monmouth Junction, NJ, United States), ALK5 inhibitor (A8301, 500 nM, Merck, Darmstadt, Germany), p38 MAPK inhibitor (SB202190, 1 μM, Merck, Darmstadt, Germany), FGF7 (100 ng/ml, R&D Systems, BioTechne, Minneapolis, MN, United States), heregulin β-1 (40 ng/ml, PeproTech, Rocky Hill, NJ, United States), and nicotinamide (10 mM, Merck, Darmstadt, Germany). Organoids were removed from the 3D matrix in every 6–8 days, centrifuged at 300*g* for 5 min, and mechanically splitted by vigorous pipetting. Cell clusters were then embedded in Matrigel (25 μl). In some experiments, organoids were treated with LGK974 (250 nM, Tocris, Bristol, United Kingdom) for 5 days, and when indicated, EVs were collected for 2 days after changing the medium after the first 3 days.

### Human PDAC Organoids

The Medical Research Council of Hungary (ETT-TUKEB^1^, no. 51323-4/2015/EKU) as the national authority approved all experiments involving human PDAC samples, and informed consent was obtained from patients. PDAC organoid lines and the corresponding fibroblast cultures previously established in our research group (Zeold et al., 2020) were used in these studies. Information on clinical data, mutations, processing tumor samples, and culturing organoids were provided (Zeold et al., 2020) (org #1, #2, and #3).

### Human Bronchiolar and LUAD Organoids

The Medical Research Council of Hungary (ETT-TUKEB, nos. 52614-4/2013/EKU and 580-5/2021/EÜIG) approved the experiments with human samples, and informed consent was obtained from patients. Surgically resected tumor samples and peripheral normal lung tissues from LUAD patients were cut into two parts for organoid and fibroblast isolation and processed according to previously published protocols (Sachs et al., 2019). Briefly, they were digested with collagenase XI (Sigma, Saint Louis, MO, United States) in DMEM/F12 for 1 h at 37°C and embedded in Matrigel to establish organoid cultures. Normal bronchiolar and LUAD organoids were cultured in lung medium containing DMEM/F12 supplemented with 2% antibiotic/antimycotic mix, 1X penicillin/streptomycin and B27 supplement (Gibco, Waltham, MA, United States), 1.25 mM *N*-acetyl-cysteine (Sigma, Saint Louis, MO, United States), 10 mM HEPES buffer (Sigma, Saint Louis, MO, United States), human R-Spondin 1 (500 ng/ml, R&D Systems, BioTechne, Minneapolis, MN, United States), human noggin (100 ng/ml, PeproTech, Rocky Hill, NJ, United States), FGF-10 (100 ng/ml, PeproTech, Rocky Hill, NJ, United States), ROCK inhibitor (Y-27632, 5 μM, MedChemExpress, Monmouth Junction, NJ, United States), ALK5 inhibitor (A8301, 500 nM, Merck, Darmstadt, Germany), p38 MAPK inhibitor (SB202190, 1 μM, Merck, Darmstadt, Germany), FGF7 (100 ng/ml, R&D Systems, BioTechne, Minneapolis, MN, United States), heregulin β-1 (40 ng/ml, PeproTech, Rocky Hill, NJ, United States), and nicotinamide (10 mM, Merck, Darmstadt, Germany). For selecting *TP53* mutant LUAD organoids, a medium containing 10 μM nutlin-3 was changed every second day for 10 days (Sachs et al., 2019; Dijkstra et al., 2020). Organoids were removed from Matrigel in every 6–8 days mechanically and centrifuged at 800*g* for 5 min, and cell clusters were washed with PBS and embedded in Matrigel again (25 μl). Clinical data of the patients are summarized in Supplementary Table 1. In some experiments, organoids were treated with LGK974 (250 nM, Tocris, Bristol, United Kingdom) for 5 days. For nanoparticle tracking analysis (NTA) measurements (see below), EVs were collected for 2 days after changing the medium after the first 3 days in LGK974.

### Mouse and Human Lung Fibroblasts

Mouse and human lung samples (see *Mouse pancreas ductal and lung organoid cultures* and *Human bronchiolar and LUAD organoids* sections) were washed with PBS and cut into small pieces (<0.5 cm^3^). After washing the tissue pieces in PBS three times, they were digested with collagenase XI (Sigma, Saint Louis, MO, United States), collagenase II (Gibco, Waltham, MA, United States), and DNase I (Merck, Darmstadt, Germany) in DMEM high glucose for 1 h at 37°C. Cells and cell clusters were then washed with PBS three times and cultured in DMEM high glucose supplemented with 15% FBS, antibiotic/antimycotic mix, 1X penicillin/streptomycin, and glutamine. In some experiments, mouse lung fibroblasts were pre-treated with LGK974 (250 nM, Tocris, Bristol, United Kingdom) for 3 days before starting EV collection for 2 days.

### EV Isolation for Functional Experiments

Serum-free conditioned medium from cultured cells or chemically defined medium from organoids was collected after 2 days. The conditioned medium was serially centrifuged at 300*g* for 5 min and 2,000*g* for 20 min at 16°C to remove cells and large EVs. Samples were then centrifuged at 12,500*g* for 20 min and ultracentrifuged (UC) at 100,000*g* for 70 min at 4°C; the EV-containing pellet was resuspended in PBS and ultracentrifuged again. The EV-containing pellets were then resuspended in PBS, their EV number was assessed with NTA (see below), and they were used in functional studies. In these experiments, 2 × 10^7^ EVs in 10 μl were added to fibroblast cultures, and the organoids were treated with 2.5 × 10^7^ EVs in 5 μl.

### Nanoparticle Tracking Analysis

Cells and organoids were cultured in serum-free medium or in the chemically defined organoid media for 2 days before the experiments, respectively. EVs were collected for 48 h, and they were serially centrifuged at 300*g* for 5 min, 2,000*g* for 20 min, and 12,500*g* for 20 min. After these centrifugation steps, 100 μl supernatant was diluted to 1 ml in PBS and the concentration and size distribution of the particles were measured on a ZetaView Z-NTA instrument (Particle Metrix, Bavaria, Germany). The cell positions were scanned at 25°C in the instrument. The following camera settings were used: auto expose, gain: 28.8, offset: 0, shutter: 100, and sensitivity: 80. The videos were analyzed with a minimum area of 5, maximum area of 1,000, and a minimum brightness of 20 by the ZetaView Analyze software 8.05.10. When the EV release from different experimental groups was compared, cells were cultured under the same conditions (time, medium volume, and tissue culture dish format) and the EV concentrations were normalized to the cell number. When determining EV number for functional experiments, the ultracentrifuged EV pellet was suspended in PBS and 10 μl samples were measured in 990 μl PBS.

### Flow Cytometry and Cell Sorting

Organoids or cell line-derived spheroids were removed from Matrigel, centrifuged at 650*g* for 5 min, washed twice with PBS, and mechanically disrupted by vigorous pipetting. They were then digested with TrypLE (Gibco, Waltham, MA, United States) until they were dissociated to single cells (5–10 min). They were re-suspended in PBS containing 1 mM EDTA, 25 mM HEPES, and 1% BSA and labeled with primary antibodies for 15 min and then with secondary antibodies for 15 min on ice. Using a FACS Calibur instrument (Becton Dickinson, Franklin Lakes, NJ, United States), 10,000 events were analyzed. In some experiments, organoid cell subpopulations were sorted with a fluorescent cell sorter (Sony SH800S, Sony Biotechnology, San Jose, CA, United States) into tubes with medium or QIAzol lysis buffer (Qiagen, Hilden, Germany) to start organoid cultures or purify RNA, respectively. Cells sorted into medium were then centrifuged at 650*g* for 10 min at 4°C, and 10,000 cells were embedded in 25-μl Matrigel droplets. Starting cell numbers were identical within the same experiment.

### Detecting EVs With Anti-CD63 or Anti-CD81-Coated Beads

Conditioned media from fibroblasts, spheroids, or organoids were harvested after 2 days, and they were centrifuged at 300*g* for 5 min and 2,000*g* for 20 min. EVs were then bound to beads coated with anti-CD63 (Thermo Fisher, Waltham, MA, United States, 10606D) or anti-CD81 (Thermo Fisher, Waltham, MA, United States, 10616D) that had been blocked with 0.1% BSA (Merck, Darmstadt, Germany) for 30 min. Into 250 μl supernatant, 20 or 6 μL of the anti-CD63 or anti-CD81-coated beads were added, respectively. Beads were incubated overnight at 4°C, washed with PBS, and EVs bound to the beads were labeled with FITC-anti-CD81 or PE-anti-CD63. The percentage of positive beads was determined by a FACS Calibur instrument. For detecting mouse EVs, anti-CD81-coated beads were produced according to our previous publication (Szvicsek et al., 2019) using the Dynabeads Antibody Coupling Kit (Invitrogen, Carlsbad, CA, United States), and 1 μl of the beads was then applied to 250 μl supernatant. Mouse EVs bound to beads were detected by PE-anti-CD81 antibody. All results were normalized to cell number. The used antibodies are listed in Supplementary Table 2.

### Immunocytochemistry and Whole-Mount Immunostaining

Cell cultures were fixed in 4% paraformaldehyde (PFA) for 20 min and blocked and permeabilized in blocking buffer (PBS with 0.2% BSA, 5% FBS, and 0.3% Triton X-100). Samples were incubated with primary antibodies at 4°C overnight in blocking buffer, washed with washing buffer (0.3% Triton X-100 and 4% NaCl in PBS), and then incubated in secondary antibodies for 2 h at room temperature. Organoids or spheroids were cultured in eight-well or four-well chamber slides (Falcon), fixed in 4% PFA for 40 min, and washed with PBS. Blocking and permeabilization steps were carried out in whole-mount blocking buffer (5% FBS, 0.2% BSA, and 0.3% Triton X-100 in PBS) for 2 h at room temperature. Organoids and spheroids were incubated with primary antibodies at 4°C overnight, washed with washing buffer (0.3% Triton X-100 and 4% NaCl in PBS), and labeled with secondary antibodies for 2 h at room temperature. Samples were then mounted with ProLong Diamond Antifade Mountant countaining DAPI (Thermo Fisher, Waltham, MA, United States) and analyzed with a Leica TCS SP8 confocal microscope. Images were evaluated by the ImageJ software. The antibodies used are listed in Supplementary Table 2.

### Transmission Electron Microscopy

The EV-containing pellet after UC was washed and resuspended in 10 μl PBS. A 2 μl droplet was dried on a 300-mesh grid (Electron Microscopy Sciences, Hatfield, PA, United States). EVs were fixed with 4% glutaraldehyde for 10 min, and the grid was washed with distilled water three times. Samples were treated with 2% phosphotungstic acid and imaged with a JEM-1011 transmission electron microscope (JEOL, Tokyo, Japan) coupled to a Morada digital camera (Olympus, Tokyo, Japan), using the iTEM software (Olympus, Tokyo, Japan).

### RNA Isolation and mRNA Measurements From Cells

RNA was isolated with the miRNeasy Micro Kit (Qiagen, Hilden, Germany) following the manufacturer’s protocol in a final volume of 14 μl in water. In some experiments, cells were directly sorted into QIAzol (Qiagen, Hilden, Germany). RNA concentration was determined with a NanoDrop, and 0.5 μg RNA (in a 20 μl final volume) was reverse transcribed with the SensiFAST cDNA Synthesis Kit (Bioline, London, United Kingdom). Quantitative PCR reactions were carried out with the SensiFAST SYBR Hi-ROX Kit (Bioline, London, United Kingdom) on an ABI 7900HT Fast Real-Time PCR instrument (384-well format, 5 μl/well volume). Results were evaluated with the following formula: relative expression level = 2^–Δ*Ct*^, where ΔCt = Ct (gene of interest) – Ct (housekeeping gene). The primers used for the SYBRGreen-based qPCR method are listed in Supplementary Table 3. When the Wnt expression profile was analyzed, we marked Ct > 36 values as absent. Heatmaps were produced with *z*-score-normalized ΔCt values with the Heatmapper program.^2^

### Sequencing

cDNA was amplified with Phusion High-Fidelity DNA Polymerase (Thermo Fisher, Waltham, MA, United States) with primers for *KRAS* (annealing temperature 65°C): CCCAGGTGCGGGAGAGA and AGGCATCATCAACACCCTGT. The PCR product was isolated from 2% agarose gel, purified by the Gel Purification Kit (Macherey-Nagel, Bethlehem, PA, United States), and sequenced with the forward and reverse primers with an Applied Biosystems 3500 Genetic Analyzer instrument (Life Technologies, Carlsbad, CA, United States). Data were analyzed by the Chromas 2.6 software (Technelysium Pty Ltd., South Brisbane, QLD, Australia). The results for mutational hotspots (codon 12,13, and 61) are shown in Supplementary Table 1.

### Statistical Analysis

Student’s unpaired *t*-tests, ANOVA, Mann–Whitney *U*-test, or Kruskal–Wallis with Dunn *post hoc* test were applied with ^∗^*p* < 0.05, ^∗∗^*p* < 0.01, and ^∗∗∗^*p* < 0.005 significance levels. When analyzing fold change data, they were log10 transformed, and these transformed data were applied to one-sample *t*-test. We used Microsoft Excel, SPSS version 25, and GraphPad softwares for statistical evaluation. Mean + SD or median and 25th and 75th percentile values for boxplots are shown.

## Results

### Wnt Secretion Is Coupled to Extracellular Vesicle Release in Normal Mouse Pancreatic Ductal Cells but Uncoupled in a Human PDAC Model

**FIGURE 1:**
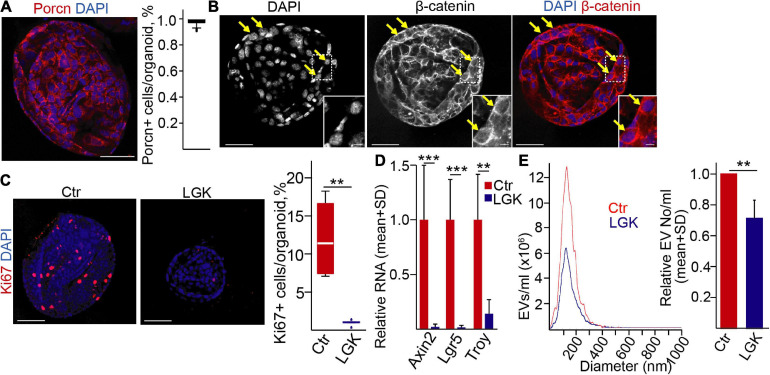
Wnt secretion affects extracellular vesicle release in normal mouse pancreatic ductal (PD) organoids. **(A)** Immunostaining for porcupine (PORCN) and quantification of confocal microscopic images. **(B)** β-catenin detection in PD organoids (confocal microscope). The arrows mark cells with nuclear β-catenin. **(C)** Immunostaining for the proliferation marker Ki67 in the presence (LGK974) or absence (Ctr) of a PORCN inhibitor. Representative confocal microscopic images (left panels) and their quantification. **(D)** The relative RNA levels of *Axin2*, *Lgr5*, and *Troy* after treatments with LGK974 (RT-qPCR, *n* = 6). Data normalized to *Hprt1* housekeeping were compared to the untreated control. **(E)** Representative images of nanoparticle tracking analysis (NTA) measurements (left panel) and their quantification (right panel, *n* = 5). Data were normalized to 10^6^ cells and then compared. For panels **(A,C)**, 15–20 images from three experiments were analyzed. Kruskal–Wallis with Dunn *post hoc* test **(C)**, *t*-test **(D)**, or one-sample *t*-test **(E)** were used with **p < 0.01, and ***p < 0.005. Scale bars: 50 μm **(A–C)** or 10 μm [**(B)** magnified panels].

Previously, we found that the percentage of Wnt-responding cells and EV secretion were closely coupled in the intestine (Szvicsek et al., 2019). To study whether this was a general phenomenon valid for other cell types as well, we first used mouse pancreatic ductal organoids. Importantly, we have already proven their ductal identity and their EV secretion (Zeold et al., 2020). Interestingly, the vast majority of the organoid cells were positive for Porcn, a key enzyme that palmitoylates Wnt proteins and that is critical in their secretion (Komekado et al., 2007; Figure 1A). Furthermore, we observed nuclear β-catenin in some cells (Figure 1B), showing the activity of the Wnt pathway. Blocking PORCN inhibits the post-translational modification of Wnt proteins, leading to the decreased secretion and activity of Wnt ligands (Wang et al., 2013). In line with these data, applying the PORCN inhibitor LGK974 diminished the proportion of the Ki67+ proliferating cells (Figure 1C) but had no effect on the active caspase-3+ apoptotic cells (Supplementary Figure 1A). Furthermore, the inhibitor resulted in the lower RNA level of the Wnt targets *Lgr5*, *Axin2*, and *Troy* (Figure 1D). Importantly, the PORCN inhibitor reduced the number of EVs in the supernatant of the organoids, measured by NTA (Figure 1E).

**FIGURE 2:**
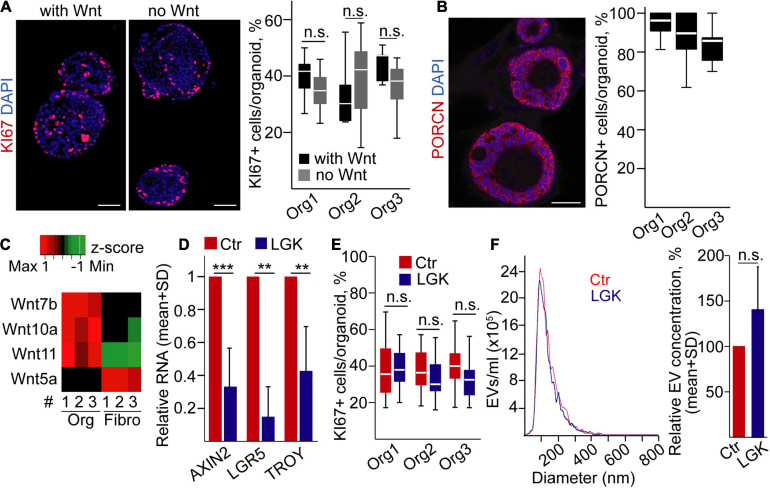
Wnt secretion is uncoupled from cell proliferation and EV release in human pancreatic ductal adenocarcinoma (PDAC) organoids. **(A)** Immunostaining for the proliferation marker KI67 and quantification of the confocal microscopic images for three organoid lines. Representative images are shown from organoid #1. In some experiments, organoids were cultured with or without recombinant Wnt3a (with Wnt and no Wnt, respectively, 100 ng/ml). **(B)** Confocal microscope analysis of organoids immunostained for Porcn and quantification of the images. **(C)** The expression level of the indicated Wnt genes in PDAC organoids and fibroblasts (RT-qPCR, *n* = 3). Data normalized to housekeeping were *z*-score transformed. Red and green colors indicate higher and lower RNA levels, respectively. The black color shows the lack of expression (Ct > 36). **(D)** Fold change of RNA levels of *AXIN2*, *LGR5*, and *TROY* (RT-qPCR). Results from samples cultured with LGK974 and normalized to *HPRT1* were compared to the untreated controls (*n* = 3 from three organoid lines). **(E)** The percentage of KI67 + cells in the organoids with or without LGK974 (analysis of confocal microscopic images). **(F)** Representative images of NTA measurements (left panel) and their quantification (right panel, normalized to 10^6^ cells, *n* = 5) in control samples and after treatment with LGK974. For panels **(A,B,E)**, experiments were repeated three times for each organoid line. Mann–Whitney *U*-test **(A,E)**, paired *t*-test **(D)**, or one-sample *t*-test **(F)** were used with **p < 0.01 and ***p < 0.005. *p* > 0.05 was considered non-significant. Scale bars: 50 μm **(A,B)**.

Next, we focused on PDAC, a cancer type of ductal origin, and we used our previously published human PDAC organoid lines that carried *TP53* mutations (Zeold et al., 2020). During PDAC progression, tumor cells may become independent of the external Wnt ligands (Seino et al., 2018). To decide whether our organoids belong to the Wnt-dependent or -independent group, we cultured them with/without Wnt3a, a prototypic canonical Wnt protein (Seino et al., 2018). Importantly, all these organoids grew even in the absence of exogenously added Wnt3a protein (Figure 2A), and they had a uniform PORCN expression (Figure 2B). They expressed Wnt ligands that had been published to be characteristic for PDAC cells, such as Wnt7a, Wnt10a, and Wnt11, at a higher level compared to PDAC patient-derived fibroblasts (Figure 2C), showing that PDAC organoids are able to produce Wnt ligands for themselves. In addition, we could not detect the RNA of the stromal Wnt5a gene (Seino et al., 2018) in PDAC organoids (Figure 2C and Supplementary Table 4). As expected, using the Wnt secretion inhibitor LGK974 resulted in a decreased expression of the Wnt targets *AXIN2*, *LGR5*, and *TROY* in PDAC organoids (Figure 2D). However, in line with a previous report showing that the presence of specific mutations, such as *TP53*, proliferation of the PDAC organoid cells resistant to the deprivation of Wnt proteins (Seino et al., 2018), we found no change in the percentage of KI67+ cells and EV secretion after treatment with LGK974 (Figures 2E,F). Similarly, we found no difference when counting the percentage of apoptotic cells, detected by immunostaining for active caspase-3 (Supplementary Figure 1B). Collectively, whereas inhibition of the activity of Wnt proteins has a critical effect on cell proliferation and EV secretion in normal pancreas ductal organoids, these processes are uncoupled in PDAC.

### Modulating the Percentage of Proliferating Cells Modifies EV Release in Mouse Bronchiolar Organoids

**FIGURE 3:**
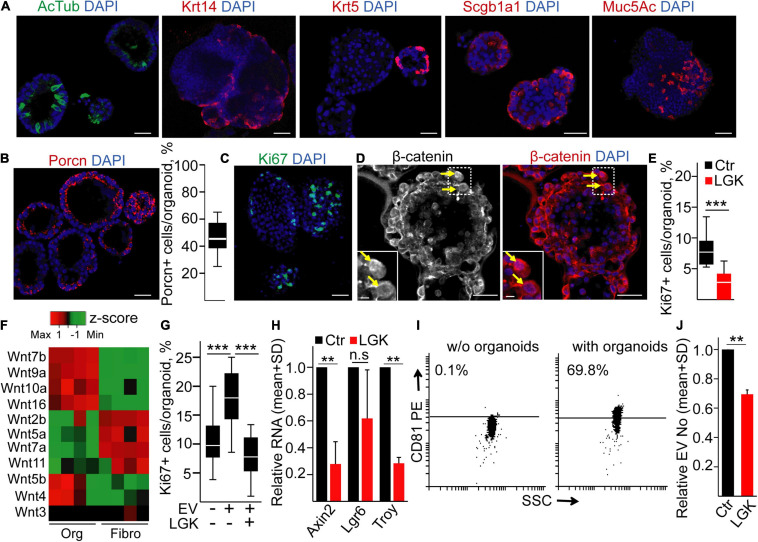
Inhibiting Wnt secretion results in a lower proliferation and EV secretion in mouse lung bronchiolar organoids. **(A)** Confocal microscopic images of organoids immunostained for acetylated tubulin (AcTub), Krt14, Krt5, Scgb1a1, and Mucin5Ac (Muc5Ac). **(B)** Immunostaining of Porcn and the quantification of PORCN + cells in the organoids. **(C,D)** Detecting the Ki67+ proliferating cells and organoid cells with nuclear β-catenin (arrows). **(E)** The percentage of Ki67+ cells in control organoids (Ctr) or after applying the PORCN inhibitor LGK974 (LGK). **(F)** The expression level of the indicated Wnt genes in organoids and in mouse lung fibroblasts (RT-qPCR, *n* = 4). Data normalized to *Hprt1* housekeeping were *z*-score transformed. Note that red and green colors indicate higher and lower RNA levels, respectively. **(G)** The percentage of proliferating cells when organoids were treated with/without lung fibroblast-derived EVs for 4 days. In some experiments, fibroblasts were pre-treated with LGK974 (LGK) 3 days before starting EV collection. **(H)** RNA fold changes in the organoids (compared to untreated controls) after treatment with LGK974 for *Axin2*, *Lgr6*, and *Troy* (RT-qPCR, *n* = 4). **(I)** Percentage of anti-CD81-coated positive beads after incubation in serum-free medium (w/o organoids) or in the conditioned medium derived from organoids (with organoids). Positive beads were detected with anti-CD81 (representative flow cytometry images). **(J)** Relative EV number after treatment of the organoids with LGK974 (NTA, *n* = 4). Data were normalized to 10^6^ cells before comparisons. For panels **(B,E,G)**, data were collected from three experiments and 13–15 images were analyzed. Mann–Whitney *U*-test **(E)**, Kruskal–Wallis and Dunn tests **(G)**, and one-sample *t*-test on log10-transformed fold change data **(H,J)** were used with **p < 0.01, ***p < 0.005. Scale bars: 50 μm **(A–D)** and 10 μm [**(D)**, magnified panels].

To further test the role of the Wnt pathway in EV secretion in another tissue, we established bronchiolar organoids from mice. They contained all bronchiolar cell types, including ciliated cells (acetylated tubulin, AcTub+), secretory cells (Mucin5Ac, Muc5Ac+), basal cells (cytokeratin-5, CK5+ or cytokeratin-14, CK14+), and club cells (Scgb1a1+) (Figure 3A). Thus, these organoids represented a good model of the *in vivo* tissue environment. To prove the presence of Wnt-producing cells, we applied immunostaining for Porcn, and we observed a heterogeneity for Porcn positivity among cells (Figure 3B), suggesting that not all cells are able to secrete active Wnt. In addition, only a subpopulation of the cells showed the proliferation marker Ki67 (Figure 3C), and some cells contained nuclear β-catenin (Figure 3D), proving the activation of the Wnt signaling pathway. Interestingly, blocking Wnt secretion by the porcupine inhibitor LGK974 decreased the number of Ki67 + cells (Figure 3E) without affecting the number of apoptotic cells (Supplementary Figure 1C), suggesting that organoid cell subpopulations critically contribute to establishing the special microniche for proliferating cells.

Fibroblasts are an abundant cell type around epithelial tissues. Interestingly, mouse organoid cells and fibroblasts had a characteristic and only partially overlapping Wnt expression pattern (Figure 3F and Supplementary Table 4), raising the possibility that fibroblasts are also critical in shaping the Wnt-dependent organoid cellular heterogeneity. Importantly, EVs are a tool for transmitting Wnt activity (Gross et al., 2012; Saha et al., 2016). In support of this hypothesis, we found that lung fibroblast-derived EVs increased the number of proliferating cells in the organoids (Figure 3G). Since there are many Wnt ligands with largely overlapping functions, we blocked the secretion of Wnt ligands in fibroblasts with LGK974, and this prevented the effect of EVs on organoid cell proliferation (Figure 3G). Of note, we proved the presence of EVs in cell culture conditioned media by anti-CD81-coated beads and NTA (Supplementary Figures 2A,B). LGK974 had no effect on either the proportion of Ki67+ proliferating or active caspase-3+ apoptotic fibroblasts (Supplementary Figures 2C,D), and it did not modify EV release from these cells (Supplementary Figure 2E). Thus, both Porcn+ organoid cells and Wnt activity coupled to fibroblast-derived EVs play a critical role in shaping the proliferating microniche in the organoids.

Modifying the microniche by applying LGK974 in organoids resulted not only in a decreased proliferation but also in a reduced RNA level of *Axin2* and *Troy* that are Wnt targets characteristic for the lung (Sachs et al., 2019; Figure 3H). Furthermore, we detected CD81+ EVs in the conditioned medium of the organoids (Figure 3I). Importantly, the lower percentage of Ki67+ cells after treatment was coupled to a decreased EV release from organoids, detected by NTA (Figure 3J). Thus, these results show that modulating specifically Wnt secretion or Wnt activity critically modifies the number of proliferating cells, and it is coupled to the change in EV release in mouse bronchiolar organoids.

### EV Secretion Intensity Is Dependent on the Proportion of Proliferating Cells in Human Bronchiolar Organoids

**FIGURE 4:**
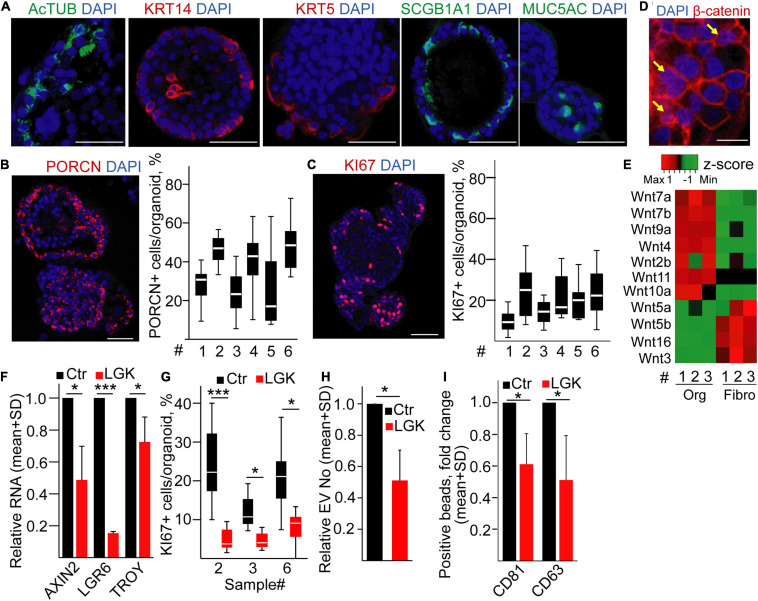
Inhibiting PORCN results in a reduced EV secretion in human bronchiolar organoids. **(A–C)** Immunostaining of human bronchiolar organoids for the indicated molecules (confocal microscopic images). For panels **(B,C)**, quantification of the images is shown for different patient-derived organoids (^#^, see Supplementary Information). **(D)** Immunostaining of β-catenin (confocal microscopic image). Arrows indicate the nuclear localization of β-catenin. **(E)** Expression profile of selected Wnt genes in the organoids and fibroblasts (RT-qPCR, matched samples were used). Results normalized to *HPRT1* housekeeping were *z*-score transformed. Red and green colors indicate higher and lower RNA levels, respectively. **(F)** Relative RNA level of *AXIN2*, *LGR6*, and *TROY* after treating organoids with LGK974 (RT-qPCR, *n* = 3). Data were normalized to *HPRT1* housekeeping and then compared to untreated samples (Ctr). **(G)** The percentage of KI67+ cells in the organoids with/without treatment with the PORCN inhibitor (confocal microscopic images were quantified). **(H)** EV number change in the presence of LGK974 (NTA, *n* = 4). Data normalized to 10^6^ cells were compared to the untreated control (Ctr). **(I)** Fold change in the percentage of anti-CD63 and anti-CD81-coated beads incubated in the conditioned medium derived from untreated (Ctr) or treated (LGK) organoids and detected with anti-CD63 or anti-CD81 antibody, respectively. Note that medium control (without EVs) was always <1%. All data were normalized to 10^6^ cells before comparisons (flow cytometry, *n* = 5). One-sample *t*-test after log10 transformation of fold change data **(F,H,I)** or Mann–Whitney test for within-sample comparisons **(G)** was used with *p < 0.05 and ***p < 0.005. Scale bars: 50 μm **(A–C)** or 20 μm **(D)**.

To test whether the percentage of proliferating cells determines the amounts of released EVs in humans, we established bronchiolar organoids from the lung tissue. Importantly, the organoid lines could be maintained for >2 months in cultures (Supplementary Figure 3A), and they contained KRT5+, KRT14+, MUC5A+, SCGB1A1+, and AcTUB+ cells, proving the presence of the major bronchiolar cell types (Figure 4A; Zhou et al., 2018; Sachs et al., 2019). We found a heterogeneity in PORCN expression among cells (Figure 4B), and we observed that only a subpopulation of the organoid cells was positive for KI67 (Figure 4C). In addition, β-catenin showed a nuclear localization in some cells (Figure 4D), indicating active Wnt signaling. When comparing the Wnt expression profile of organoid cells and fibroblasts isolated from normal lung tissue, we found that a Wnt gene group was characteristic for the epithelial cells (Figure 4E and Supplementary Table 4), thus showing that bronchiolar organoids are able to produce Wnt ligands for themselves. In line with these data, inhibiting Wnt secretion decreased the RNA levels of the Wnt targets *AXIN2*, *LGR6*, and *TROY* and the percentage of KI67+ cells in the organoids (Figures 4F,G) without changing the proportion of apoptotic cells (Supplementary Figure 3B). Of note, modulating the size of the active Wnt-producing cell population resulted in a change in EV secretion (Figures 4H,I). Importantly, we proved the EV identity of these particles with immune adsorption on antibody-coated beads and by transmission electron microscopy (TEM) (Supplementary Figures 3C,D). Thus, our data provide evidence that the size of Wnt-producing cell subpopulation is coupled to an increased EV release in normal human bronchiolar organoids.

### LUAD Organoids Contain a Wnt-Producing Microenvironment That Affects EV Release

Although the intratumoral heterogeneity, establishing Wnt-producing and Wnt-responding cells, is critical in a mouse model of LUAD for the progression of the disease (Tammela et al., 2017), the presence of such a microenvironment in humans has not yet been directly proven. To study the role of the Wnt-producing microenvironment on EV secretion in LUAD, we first used commercially available cell lines as a standard system to model cell biological processes in tumors. To test whether they show a heterogeneity for Wnt production, we cultured cell lines of primary NSCLC origin (A549 and H1975) and control lung epithelial cells (BEAS-2B) in 3D conditions. Interestingly, we found no massive positivity for PORCN in any of the studied cell line-derived spheroids (Supplementary Figure 4A). Accordingly, the addition of LGK974 did not modify the percentage of KI67+ cells and EV release in any of the 3D cell lines either (Supplementary Figures 4B,C).

**FIGURE 5:**
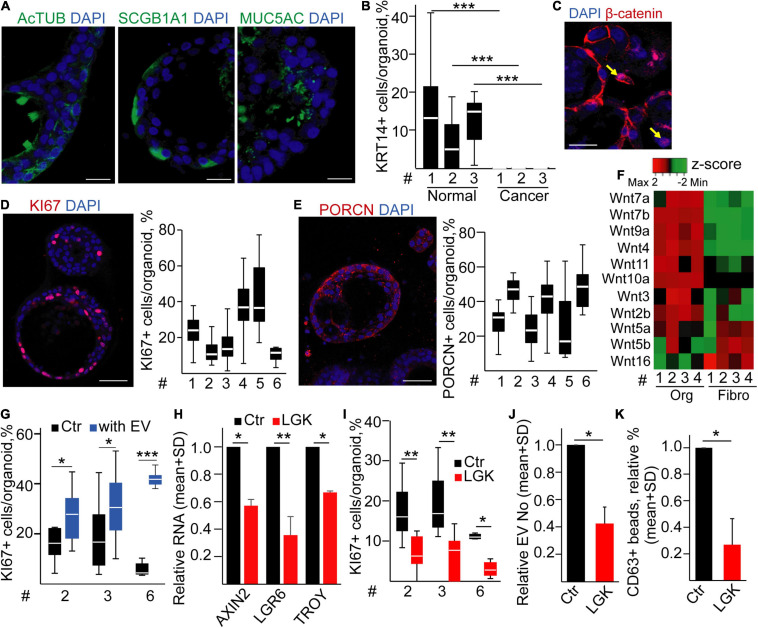
Modulating the proliferative microniche in human lung adenocarcinoma (LUAD) organoids leads to a reduced EV release. **(A)** Detection of AcTUB, SCGB1A1, and MUC5AC in LUAD organoids (immunostaining and confocal microscopic images). **(B)** The percentage of KRT14+ cells in three matched sample pairs representing bronchiolar and LUAD organoids (immunostaining and confocal microscopic analysis, 10 images/sample). **(C)** Confocal microscopic image for β-catenin. The arrows indicate nuclear localization. **(D,E)** Representative images and their quantification for KI67 **(D)** and PORCN **(E)** from different patient-derived LUAD organoids. **(F)** Expression profile of selected Wnt genes in LUAD organoids and fibroblasts (RT-qPCR, matched samples were used). Results normalized to *HPRT1* housekeeping were *z*-score transformed. Red and green color codes indicate higher and lower RNA levels, respectively. **(G)** The ratio of KI67+ proliferating LUAD organoid cells in the absence (Ctr) and presence of LUAD fibroblast-derived EVs for 4 days (with EV). Confocal microscopic images were evaluated. **(H)** Relative RNA for the indicated genes after LGK974 treatment, compared to the untreated controls (Ctr) (RT-qPCR, *n* = 3). **(I)** The percentage of proliferating cells in the organoids in the presence or absence of LGK974 (quantification of confocal microscopic images). **(J)** EV number changes after adding the PORCN inhibitor (LGK), measured with NTA (*n* = 4). **(K)** Changes in the percentage of anti-CD63-coated beads, incubated in media from control or LGK974-treated LUAD organoids. Data were normalized to 10^6^ cells before determining fold changes (flow cytometry, *n* = 4). For panels **(D,E,I)**, 14–16 images from two experiments were collected for each organoid line. Mann–Whitney *U*-test **(B,G,I)** and one-sample *t*-test **(H,J,K)** were applied with *p < 0.05, **p < 0.01, and ***p < 0.005. Scale bars: 20 μm **(A,C)** or 50 μm **(D,E)**.

Since cell line-derived spheroids lacked PORCN expression, we next isolated tumor organoids from patients according to previous methods (Sachs et al., 2019). Tumor-derived samples give rise not only to organoids of cancer origin but also to normal organoids as well that often overgrow the tumor organoids (Dijkstra et al., 2020). *TP53* is the most frequently mutated gene in LUAD (Cancer Genome Atlas Research Network, 2014). Thus, we applied nutlin-3, resulting in p53 stabilization, cell cycle arrest, and ultimately death of cells with wild-type p53 (Sachs et al., 2019; Dijkstra et al., 2020). Whereas none of the normal lung organoid cultures survived nutlin-3 treatment, many of the LUAD samples contained surviving organoids after this selection step, and they could be cultured for >2 months. We used only organoids resistant to nutlin-3 as LUAD samples (Supplementary Figures 5A–C) (Dijkstra et al., 2020). Interestingly, organoids contained cells with differentiation markers, such as AcTUB+, MUC5AC+, or SCGB1A1+ cells (Figure 5A). Importantly, LUAD organoids contained only a marginal number of KRT14+ cells compared to normal lung organoids (Figure 5B) that is a previously published hallmark of organoids of tumor origin (Dijkstra et al., 2020). Furthermore, we observed the nuclear localization of β-catenin in some LUAD cells (Figure 5C), and LUAD organoids displayed a cellular heterogeneity for KI67 and PORCN (Figures 5D,E), showing the presence of a Wnt-producing microniche in human LUAD as well. To study how the size of the proliferating LUAD cell population is regulated, we established fibroblast cultures from the tumor of LUAD patients (LUAD-F). These cultures displayed a heterogeneity for the widely accepted fibroblast marker αSMA (Supplementary Figure 6A). Of note, LUAD cells and fibroblasts had a characteristic and only partially overlapping Wnt expression pattern (Figure 5F and Supplementary Table 4), showing that both cell types are able to produce Wnt ligands. Importantly, LUAD-Fs secreted EVs, detected with antibody-coated beads and NTA (Supplementary Figures 6B,C), and these EVs increased the number of proliferating cells in LUAD organoids (Figure 5G).

To further characterize the Wnt-producing organoid microniche, we determined whether proliferating and PORCN+ cells accumulate in some specific cell subpopulations. Whereas we observed a higher percentage of KI67+ and PORCN+ cells within the AcTUB+ cell population when comparing to other cell type-specific markers in normal human bronchiolar organoids, we detected a more even distribution of the proliferating and Wnt-secreting cells among AcTUB+ and MUC5AC+ cells in LUAD samples (Supplementary Figures 7A,B). Interestingly, PORCN was expressed in a large percentage of SCGB1A1 tumor cells, too (Supplementary Figure 7B). Thus, these data show that PORCN+ and proliferating cells are not coupled to specific cell markers in LUAD that can be explained by the lack of terminal differentiation of tumor cells to specific cell types.

We next focused on the effect of the PORCN+ organoid microniche. Similarly to the normal human bronchiolar organoids, we found that blocking Wnt secretion resulted in a reduced level of *AXIN2*, *LGR6*, and *TROY* RNAs (Figure 5H) and in a decreased proliferation (Figure 5I) without modifying the percentage of apoptotic cells (Supplementary Figure 8A). Of note, applying PORCN inhibitor led to a reduced EV release (Figures 5J,K). Importantly, we proved the presence of EVs in the organoid-derived culture medium by antibody-coated beads and TEM (Supplementary Figures 8B,C). Thus, our results indicate that the cellular heterogeneity established by both intra-tumoral Wnt-producing cells and fibroblast-derived EVs critically determines both cell proliferation and the EV secretion intensity in LUAD organoids.

### The CD133^*high*^ LUAD Cell Population Gives Rise to Organoids Enriched in PORCN+ Cells and a High EV Release

**FIGURE 6:**
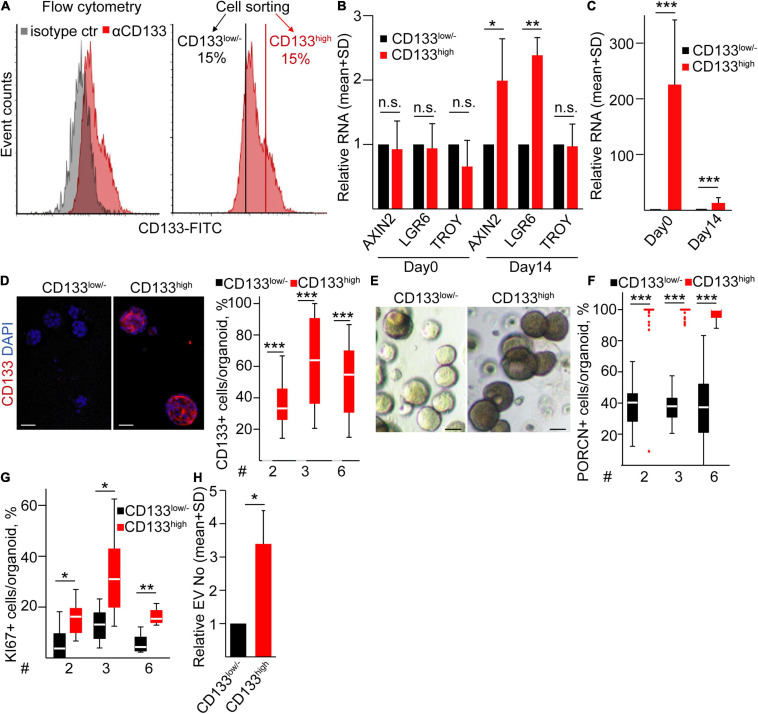
CD133^*high*^ LUAD cells produce organoids with a high proliferation rate and EV release. **(A)** CD133 expression on LUAD organoid cells and the strategy for sorting CD133^*high*^ and CD133^*low/–*^ cells (flow cytometry and cell sorting). **(B)** Relative RNAs for *AXIN2*, *LGR6*, and *TROY* from freshly sorted cells (day 0) or from organoids initiated by CD133^*high*^ or CD133^*low/–*^ cells, measured on day 14 (RT-qPCR, *n* = 4). Data were normalized to *HPRT1* housekeeping, and RNA levels of the CD133^*high*^ population were then compared to the CD133^*low/–*^ results in each sorting experiment. **(C)** Relative RNA level of *CD133* from the sorted cells (day 0) and organoids initiated by these cells (day 14). Normalized RNA level from the CD133^*low/–*^ population was taken one (RT-qPCR, *n* = 4). **(D)** Immunostaining for CD133 and the quantification of confocal microscopic images in organoids derived from sorted cells (CD133^*low/–*^ or CD133^*high*^) on day 14 (*n* = 3). **(E)** Bright-field microscopic images of organoids initiated by the indicated sorted LUAD cell populations. **(F,G)** Percentage of PORCN + **(F)** and KI67+ **(G)** cells in the organoids derived from sorted CD133^*low/–*^ and CD133^*high*^ cells on day 14. **(H)** Relative EV number from CD133^*low/–*^ or CD133^*high*^ cell-derived organoids on day 14 (NTA, *n* = 4). Data were normalized to 10^6^ cells before comparisons. Data for three LUAD samples are shown in panels **(D,F,G)** with 10–15 images analyzed from three replicates. One-sample *t*-test **(B,C,H)** or Mann–Whitney *U*-test **(D,F,G)** were used with *p < 0.05, **p < 0.01, and ***p < 0.005. Scale bars: 50 μm.

Previous studies suggested that CD133 marks an aggressive cell population in lung cancer (Lundin and Driscoll, 2013) that may be explained by the differential ability of tumor cell subpopulations to form a PORCN+ microniche. To study whether this cell population has a different ability to create the Wnt-producing microniche compared to other LUAD cells, we fluorescently sorted CD133^*high*^ and CD133^*low/*–^ cells from LUAD patient-derived organoids (Figure 6A). We found no difference in the Wnt targets *AXIN2*, *LGR6*, and *TROY* between the freshly sorted cells (Figure 6B), showing that the general Wnt intensity does not differ between these two subpopulations. Importantly, CD133^*high*^ cells formed bigger organoids, and these organoids had a higher *CD133* RNA level and more CD133+ cells even after 2 weeks compared to organoids from CD133^*low*/–^ cells (Figures 6C–E). Thus, organoids maintain the difference in the CD133 expression pattern. CD133^*high*^ organoids had an elevated Wnt activity, indicated by the higher RNA level of *AXIN2* and *LGR6* (Figure 6B). Surprisingly, in contrast to CD133^*high*^ organoids where all cells were positive for PORCN, only a subpopulation of CD133^*low*/–^ LUAD organoid cells expressed this enzyme (Figure 6F), suggesting that not all cells are able to secrete active Wnt ligands. The functional consequence of the difference in the size of the Wnt-producing cell population was reflected by the lower number of KI67+ proliferating cells in CD133^*low*/–^ cell-derived organoids as well (Figure 6G). Thus, all these data suggest that the more PORCN + cells in CD133^*high*^ cell-derived organoids lead to an increased number of proliferating cells and Wnt activity within the organoids. In addition, NTA measurements indicated a higher EV concentration in the supernatants of CD133^*high*^ LUAD organoids (Figure 6H) that contained more proliferating cells. Collectively, in contrast to CD133^*high*^ organoids where the majority of cells both produced and responded to Wnt with proliferation, the CD133^*low*/–^ organoids showed a heterogeneity for Wnt secretion. This resulted in a lower proliferation ratio and EV secretion intensity as compared to CD133^*high*^ cell-derived organoids.

## Discussion

In this study, we provide evidence that Wnt activity and cell proliferation are coupled to an elevated secretion of EVs in organoids of various tissue origins. Furthermore, blocking Wnt secretion by PORCN inhibitors resulted in a lower cell proliferation and in a diminished EV release in pancreatic ductal, lung bronchiolar, and LUAD organoids. Interestingly, whereas the inhibitors of Wnt secretion resulted in a decrease of the RNA levels of Wnt targets, they had no effect on the proliferation rate in PDAC organoids and, accordingly, the EV secretion was not altered either. In addition, we detected a Wnt secretory tumor cell subpopulation in LUAD organoids, thus proving the presence of a Wnt-producing micro-environment not only in mouse lung cancer (Tammela et al., 2017) but also in a human model. Importantly, fibroblast-derived EVs induced cell proliferation in normal bronchiolar and LUAD organoids via Wnt activity, highlighting the importance of fibroblast-derived EVs in establishing the cellular heterogeneity. Furthermore, CD133^*high*^ expression marked a LUAD cell population with the ability to produce organoids with more PORCN+ and proliferating cells compared to CD133^*low/*–^ cells.

Most studies focusing on the intensity of EV release used only one model or classical 2D cell cultures that do not reflect the cellular heterogeneity. For example, drugs that interfere with cellular metabolism influenced EV release from cancer cell lines (Wen et al., 2020), bystander T cells induced EV release from dendritic cells (Lindenbergh et al., 2019), and hypoxia is an important inducer of EV release both in cancers and in non-neoplastic cells (King et al., 2012; Kucharzewska et al., 2013; Zhang et al., 2017). However, organoids are considered as a superior method to model the cellular heterogeneity within the tissues of epithelial origin (Sasaki and Clevers, 2018). Based on this feature of organoids, they have been successfully applied to uncover disease mechanisms; the role of mutations in tumorigenesis, and the mechanisms of tumor progression, developmental processes, etc. (Fiorini et al., 2020; Fujii and Sato, 2021). Most organoid types critically depend on either endogenously produced or exogenously supplemented Wnt ligands (Schutgens and Clevers, 2020). Furthermore, organoid cultures identified the central role of Wnt signaling in shaping the histologic variation in diffuse gastric cancer (Togasaki et al., 2020). Thus, the Wnt pathway is important not only in the differentiation of cell types and morphogenesis but also in producing a proliferating microniche in cancers, leading to cellular heterogeneity. Since PORCN is critical in the palmitoylation of Wnt proteins, PORCN-deficient cells are unable to secrete Wnt proteins (Barrott et al., 2011), and PORCN inhibitors are widely used tools to block Wnt secretion (Koo et al., 2015; van de Wetering et al., 2015). In line with these data, we found that PORCN inhibitors decreased the expression of Wnt targets and cell proliferation in different organoids, probably in an autocrine and/or paracrine manner. Importantly, PORCN was shown to mark Wnt-producing tumor cells in a mouse model of LUAD that created a special microenvironment for Wnt-responding cancer cells. Furthermore, the size of this cell population critically determined disease survival (Tammela et al., 2017). In our studies, we provide human evidence for the existence of this microenvironment in LUAD and we show that organoids can model this microniche.

The cell–cell communication via EVs has recently attracted much interest, and tumor cell-derived EVs have been suggested to exert widespread effects such as coagulation, vascular leakiness, reprogramming of stromal recipient cells to support the pre-metastatic niche formation, etc. (Becker et al., 2016; Maia et al., 2018). Their sometimes opposing roles in lung cancer progression are indicated by studies showing that EVs modulated integrin trafficking in fibroblasts and enhanced tumor cell migration in NSCLC by carrying podocalyxin (Novo et al., 2018), and they prevented metastatic angiogenesis in LUAD by transporting miR-192 (Valencia et al., 2014). Similarly to CRC (Szvicsek et al., 2019; Oszvald et al., 2020), we found that fibroblast-derived EVs induced cell proliferation both in bronchiolar and in LUAD organoids, thus proving the importance of fibroblast-derived EVs and that this mechanism is not restricted to CRC.

Genetically engineered PDAC organoids with *TP5*3 and *CDKN2A* mutations survived and slowly expanded in the Wnt-free culture condition, suggesting that the presence of these genetic alterations allowed organoids to circumvent the apoptosis/senescence responses induced by Wnt removal (Seino et al., 2018). In line with this, we found that our PDAC organoid lines were independent not only of exogenously added Wnt proteins but also blocking endogenous Wnt production had no effect on cell proliferation either. In contrast, in a *KRas* and *Trp5*3 mutant mouse LUAD model, inhibiting Wnt secretion led to a markedly reduced tumor growth and a prolonged survival (Tammela et al., 2017). Similarly, we found that LUAD patient-derived organoids, selected for *TP53* mutations, contained Wnt-producing cells, and their activity was important for tumor cell proliferation. Thus, PDAC patients may contain additional mutations beside *TP5*3 that are not characteristic for LUAD. Indeed, the genomic landscapes of these two tumors show only a partial overlap when comparing the list of mutated genes (Cancer Genome Atlas Research Network, 2014; Cancer Genome Atlas Research Network. Electronic address and Cancer Genome Atlas Research Network, 2017).

By using the organoid technology, we previously found that *Apc* mutation, resulting in the continous Wnt activation, led to an enhanced proliferation and EV secretion from intestinal organoids (Szvicsek et al., 2019). Here we provide evidence that Wnt secretion and the resulting higher cell proliferation rate are closely coupled to an increased EV release in normal tissue-derived and LUAD organoids as well, but not in PDAC. Wnt pathway activation is closely coupled to proliferation in most models. Mouse organoid models are frequently used to clarify scientific questions for disease mechanisms and developmental biology. Importantly, we provide here evidence that Wnt secretion, cell proliferation, and EV release are coupled not only in normal human but also in mouse, such as in pancreatic ductal and lung bronchiolar organoids as well. Since applying a PORCN inhibitor resulted in a decreased Wnt target expression without modifying proliferation and EV secretion in PDAC organoids, this raised the possibility that Wnt activation induced EV release indirectly, via cellular proliferation. Indeed, an elevated proliferation rate is characteristic for most tumors and they generally secrete more EVs compared to the original tissue. This higher EV release in cancer cells may be explained by the elevated fusion of MVBs with the plasma membrane (Bebelman et al., 2018).

Intra-tumoral cellular heterogeneity is a major hallmark of lung cancers. Several molecules, such as CD133 (Chen et al., 2008; Jia et al., 2020), have been suggested as markers of the aggressive cancer cell population with stem cell features. Importantly, although PORCN+ cells have a critical role in LUAD progression in a mouse model (Tammela et al., 2017), no attempt has been made to isolate these Wnt-producing niche cells. Here we found that sorted CD133^*high*^ LUAD organoid cells produced more PORCN+ cells as compared to the CD133^*low*/–^ subpopulation, identifying an important feature that may contribute to the aggressive behavior of the CD133^*high*^ cells. In addition, CD133^*high*^ cell-derived organoids with an elevated number of Wnt-secreting cells may explain the higher number of proliferating cells.

**FIGURE 7:**
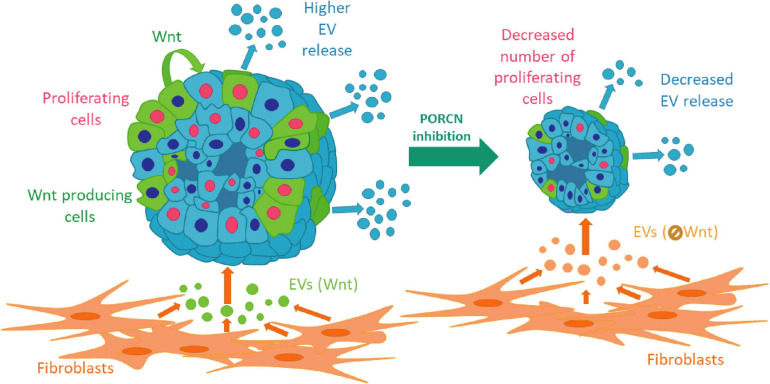
A schematic representation of our results.

Collectively, we provide evidence that intra-organoid Wnt secretion is critical for establishing a proliferation niche in normal pancreas, bronchiolar organoids, and LUAD samples. This niche is modified not only by organoid cell subpopulations but also by fibroblast-derived EVs via transmitting Wnt activity. We show that the coupling of an elevated EV release with cell proliferation, induced by Wnt activation, is characteristic for both normal and some cancer organoids, thus suggesting that this mechanism is not restricted to only one tissue type (Figure 7). Importantly, our data also indicate that EV release is regulated differentially in tumors of different origins. Since the EV-based diagnostics critically depends on both the amount and cellular source of EVs, our findings may be of critical significance for identifying the regulation of EV release in multiple tissues.

## Data Availability Statement

The raw data supporting the conclusions of this article will be made available by the authors, without undue reservation.

## Ethics Statement

The studies involving human participants were reviewed and approved by Medical Research Council of Hungary (ETT-TUKEB, No 52614-4/2013/EKU, 580-5/2021/EÜIG,51323-4/2015/EKU), H-1051 Budapest, Széchenyi István tér 7-8, Hungary. The patients/participants provided their written informed consent to participate in this study. The animal study was reviewed and approved by Pest County Government Office of Hungary (veterinary authority), No PEI/001/1781-3/2015, H-1052, Budapest, Városház u. 7, Hungary and Semmelweis University Animal Care and Use Committee, H-1089, Budapest, Nagyvárad tér 4, Hungary.

## Author Contributions

GS made significant contributions in the conception and design, collection and/or assembly of data, data analysis and interpretation, and manuscript writing. AS made significant contributions in the collection and/or assembly of data and data analysis and interpretation. AB, TT, LR, TH, and LB made significant contributions in the provision of study material or patients. PL made significant contributions in the data collection. JM made significant contributions in the provision of study material and patient data. EB made significant contributions in the data interpretation and manuscript reviewing. ZW made significant contributions in the conception and design, financial support, data analysis and interpretation, manuscript writing, and final approval of manuscript. All authors contributed to the article and approved the submitted version.

## Conflict of Interest

The authors declare that the research was conducted in the absence of any commercial or financial relationships that could be construed as a potential conflict of interest.

## Footnotes

1 https://ett.aeek.hu/en/secretariat/

2 www.heatmapper.ca
