# Supplementary material for: Wnt Activity and Cell Proliferation Are Coupled to Extracellular Vesicle Release in Multiple Organoid Models
Source: Front Cell Dev Biol. 2021 Jun 24;9:670825. doi: 10.3389/fcell.2021.670825 (PMC8264557; doi:10.3389/fcell.2021.670825)
Supplement: Supplementary file 1 [file Data_Sheet_1.PDF]

Wnt activity and cell proliferation are coupled to extracellular vesicle release in multiple organoid models

No	Age (years)	Gender	Smoking	Histology	Grade	Stage	pTNM	Neoadjuvant treatment	KRAS mutation (Codon12, 13,61)
1	60	F	never smoker	LUAD	Grade III	IIIB	T3 N2 M0	no	no
2	68	M	ex-smoker	LUAD	Grade III	IB	T2a N0 M0	no	no
3	56	F	current smoker	LUAD	Grade III	IB	T2a N0 M0	no	het c.183 A>T, Q61H
4	64	M	ex-smoker	LUAD	Grade II	IIB	T1b N1 M0	no	no
5	83	F	never smoker	LUAD	Grade II	IIA	T2b N0 M0	no	no
6	66	F	ex-smoker	LUAD	Grade III	IIB	T3 N0 M0	no	no

Table S1. Clinical parameters and *KRAS* mutations of the LUAD patients involved in our studies.

Antibody	Source	Clone/Cat No	Dilution
FITC anti-human CD81	Molecular Probes	A15753	1:25
PE anti-human CD63	Sigma	SAB4700218	1:25
PE anti-mouse CD81	Invitrogen	MA517941	1:25
anti-human CD133/2	Miltenyi Biotec	130-090-851	1:50
anti-active Caspase3	R&D Systems	AF835	1:200
anti-mouse/human β-Catenin	BD Transduction Lab.	610153	1:250
anti-mouse/human KI67	Abcam	ab16667	1:200
anti-mouse/human KI67	Invitrogen	14-5699-82	1:300
anti-mouse/human αSMA	Merck	A5228	1:200
anti-AcTUB	Santa Cruz Biotech.	sc-23950	1:100
anti-human SCGB1A1	Cloud-Clone Corp.	A20181130456	1:100
anti-mouse Scgb1a1	Santa Cruz Biotech	sc-365992	1:100
anti-mouse/human MUC5AC	Santa Cruz Biotech.	sc-21701	1:100
anti-mouse/human KRT5	BioLegend	905501	1:200
anti-mouse/human KRT14	BioLegend	905304	1:250
anti-mouse/human PORCN	Abcam	ab105543	1:300
anti-rabbit IgG Alexa 568	Invitrogen	A11011	1:500
anti-rabbit IgG Alexa 488	Invitrogen	A21206	1:500
anti-mouse IgG Alexa 488	Invitrogen	A21202	1:500
anti-mouse IgG Alexa 568	Invitrogen	A10037	1:500

Table S2. Antibodies used in our experiments.

**Primer name**	**Forward primer**	**Reverse primer**
hAXIN2	CTGGCTATGTCTTTGCACCA	CTTCACACTGCGATGCATTT
hLGR5	AGTGCTGTGCATTTGGAGTG	AGGGCTTTCAGGTCTTCCTC
hLGR6	TGACAGACAACCAGCTGACC	TGAAGAAGCTGGCACACATC
hTNFRSF19 (TROY)	GATGCACAGTGTGTGACGTG	CATGTCTTGAAAGCCGACAA
hWNT2b	TGGATGCCAAGGAGAAGAGG	CGGAAATCTGAGAGTGCACG
hWNT3	TGGAACTGCACCACCATAGA	CCCCTTATGATGCGAGTCAC
hWNT4	ACAGTCGTTTGTGGATGTGC	CCAGCACGTCTTTACCTCAC
hWNT5a	TCTGTTTTTGGCAGGGTGAT	GCGGTAGCCATAGTCGATGT
hWNT5b	TTTGGGAGAGTCATGCAGAT	TAGCCGTACTCCACGTTGTC
hWNT7a	CCGGACTCTCATGAACTTGC	ACGGCCTCGTTGTACTTGTC
hWNT7b	TCAACGAGTGCCAGTACCAG	CAGTTGCTCAGGTTCCCTTG
hWNT9a	AGACGGTCAAGCAAGGATCT	GTGCCGTCTCATACTTGTGC
hWNT10a	ACTCGCAACAAGATCCCCTA	TAAGCGGTGCAGCTTCCTAC
hWNT11	CAGGATCCCAAGCCAATAAA	TATCGGGTCTTGAGGTCAGC
hWNT16	GAAACACCACGGGCAAAGAA	TGTTTTCACAGCACAGGAGC
hHPRT	TGAGGATTTGGAAAGGGTGT	TCCCCTGTTGACTGGTCATT
mAxin2	CTCCCCACCTTGAATGAAGA	ACTGGGTCGCTTCTCTTGAA
mLgr5	CCTGTCCAGGCTTTCAGAAG	CTGTGGAGTCCATCAAAGCA
mLgr6	TGTCTCAGGCCTTCTCCAAG	GCCTCCTCTTCCTCTGGATG
mTnfrsf19 (Troy)	AGTTGCGAAACCGGAGATTG	ACCCCAGTCTTCCTTGAACC
mWnt2b	GGATGGGGCCAATTTCACAG	TCGGCCACAACACATGATTT
mWnt3	CATCTTTGGGCCTGTCTTGG	GGTGGCCCCTTATGATGTGA
mWnt4	CCGGGCACTCATGAATCTTC	GTGGCACCGTCAAACTTCTC
mWnt5a	CAACAATGAAGCAGGCCGTA	GCCGCGCTATCATACTTCTC
mWnt5b	GCCGAGCTCTCATGAACCTA	TTCTCCTTCAAACGGTCCCC
mWnt7a	TACACAATAACGAGGCGGGT	CGGCCTCGTTGTATTTGTCC
mWnt7b	TTCTCGTCGCTTTGTGGATG	ACTTAGGTAGCGTGGTCCAG
mWnt9a	ACTTCCACAACAACCTCGTG	GTGTTTTAGGTGCTTGCCCA
mWnt10a	CATCCATGAGTGCCAGCATC	CTTCAGTTTACCCAGAGCGC
mWnt11	CAGGATCCCAAGCCAATAAA	GACAGGTAGCGGGTCTTGAG
mWnt16	CCACTACCACTTCCACCCAG	GGTGTCACAGGAACATTCGG

mHprt1	GCGATGATGAACCAGGTTATGA	GCCTCCCATCTCCTTCATGA

Table S3. Primer sequences used in our studies.

Table S4. RT-qPCR results of Wnt genes from organoids and fibroblasts (see separate Excel sheets).

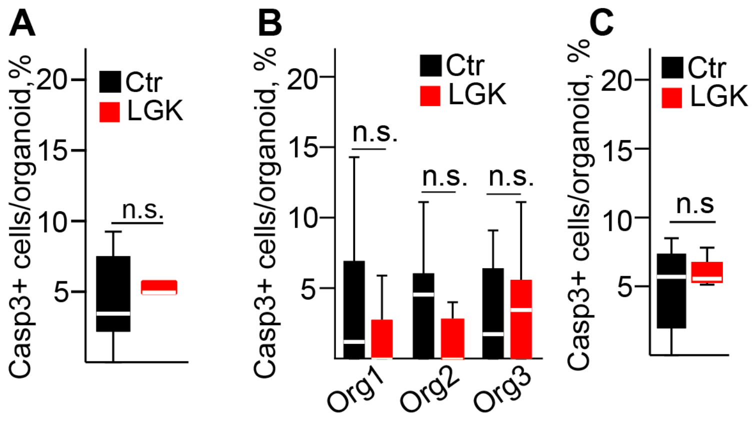

Figure S1. Blocking Porcn does not affect the apoptosis in multiple organoid models. A-C) Immunostaining for active caspase-3 of organoids cultured in the presence/absence of the Wnt secretion inhibitor LGK974 (LGK) (quantification of confocal images, n=13-15 from two replicates). Mouse pancreatic ductal (A), human PDAC (B) or mouse lung bronchiolar (C) organoids were used. Mann-Whitney U-test was used, n.s.: $p>0.05$.

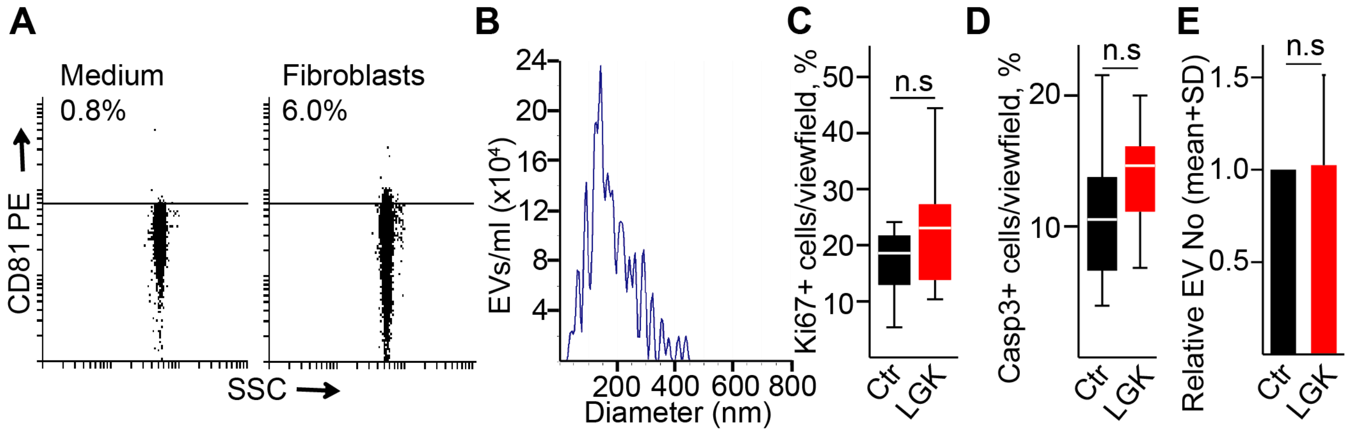

Figure S2. Blocking Porcn does not affect the proliferation, apoptosis and EV release of mouse lung fibroblasts. A) Flow cytometric image of anti-CD81-coated beads, incubated in cell-free medium (medium) or in the conditioned medium from fibroblasts (fibroblasts). EVs on the beads were detected with anti-CD81. The numbers indicate the percentage of positive beads. B) NTA histogram from the conditioned medium of a fibroblast culture after centrifuging the sample at 12,500g. C-D) The percentage of Ki67+ proliferating (C) and active caspase-3+ apoptotic (D) control (Ctr) or LGK974-treated (5 days) (LGK) fibroblasts. Data were collected from 15-20 images from three experiments. E) Relative EV numbers in LGK974-treated fibroblasts (n=3). Data were normalized to 10^6 cells prior comparing them to the untreated control (Ctr). Mann-Whitney U-test (C, D) and one-sample t-test (E) were used with $p>0.05$ as non significant.

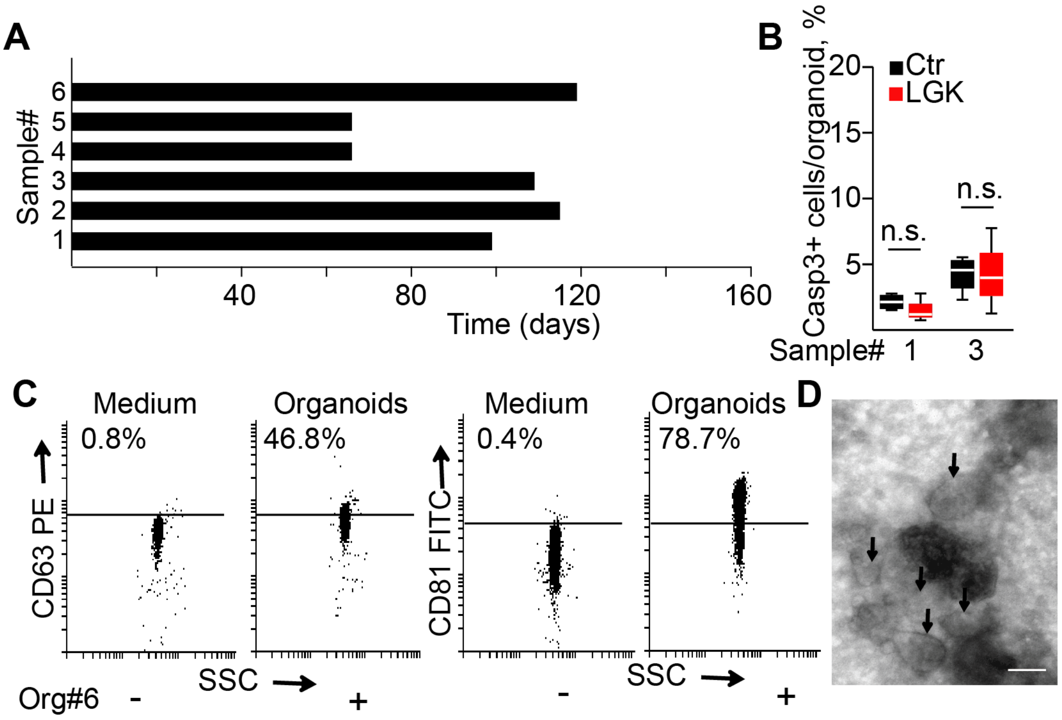

Figure S3. Human bronchiolar organoids release EVs. A) Culturing time of the organoids isolated from different patients. B) The percentage of active caspase-3+ cells in the organoids with/without LGK974 treatment (quantification of confocal images, n=13-15 from three replicates). Data were obtained from two different patient-derived organoid lines. C) Detecting EVs with anti-CD81 or anti-CD63-coated beads from the medium and from the supernatant of organoids (flow cytometry). The numbers indicate the percentage of positive beads. D) Transmission electron microscopy (TEM) image from the conditioned medium of an organoid line after ultracentrifugation. The arrows mark EVs. Mann-Whitney U-test was used (B) where $p>0.05$ was regarded non-significant (n.s.). Scale bar: 100nm (D).

A

3D	PORCN+, %	Ctr	LGK974
A549	mean	0,6	0
	SD	1,8	0
H1975	mean	3,2	1,2
	SD	3,0	1,8
BEAS-2B	mean	0	0
	SD	0	0

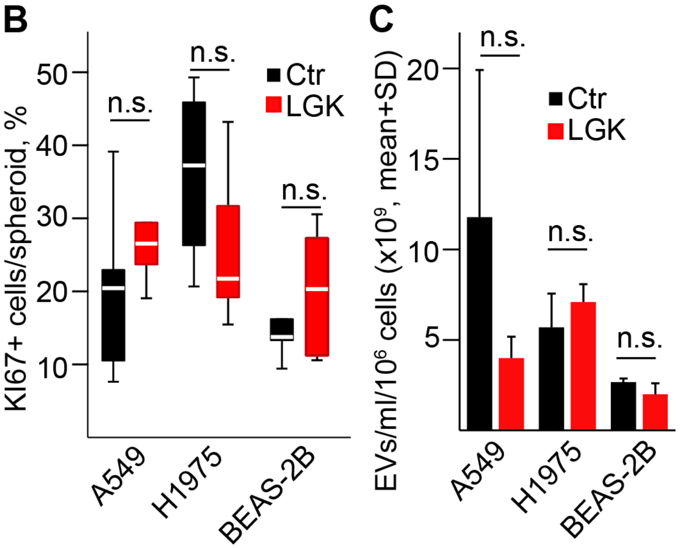

Figure S4. Human NSCLC cell line spheroids do not display heterogeneity for PORCN expression. A) Quantification of control and LGK974-treated (5 days) spheroids after immunostaining for PORCN. Confocal microscopic images were evaluated for NSCLC cell lines (A549, H1975) and for control immortalized bronchiolar cells (BEAS-2B). Note the low values for all cell lines and experimental conditions. B) The percentage of KI67+ proliferating cells in control and treated (LGK974) NSCLC and bronchiolar cell lines. C) NTA measurements from the indicated cells (n=3). Twenty images from three experiments were evaluated in A and B. Mann-Whitney U-test (B) or t-test (C) were used where p>0.05 was regarded non-significant.

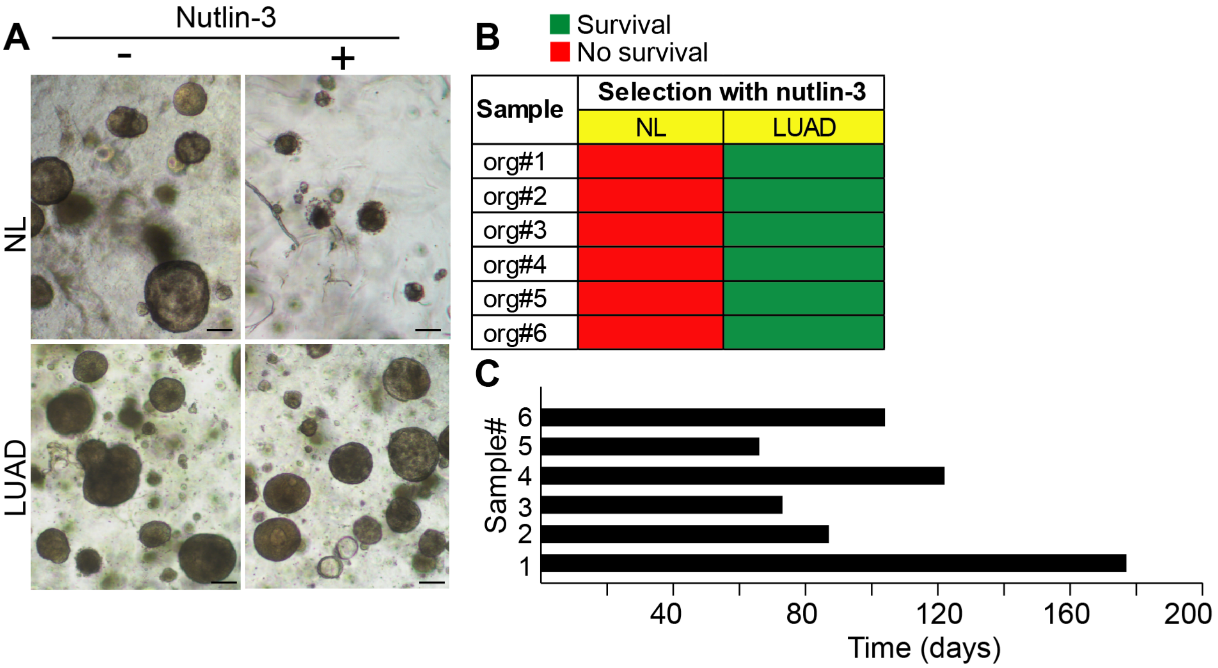

Figure S5. Only LUAD, but no normal lung bronchiolar (NL) organoids survive nutlin-3 selection. A) Bright field images of NL and LUAD organoids in the presence or absence of nutlin-3 (10µM for 10 days). B) Survival pattern of NL and LUAD organoids, isolated from the indicated patients (see Supplementary Information). Bright field images were evaluated after 10 days with nutlin-3 and they were compared to samples without nutlin-3 as positive control. C) Culturing times of patient-derived LUAD organoids after selection with nutlin-3 (10µM for 10 days). Scale bars: 50µm (A).

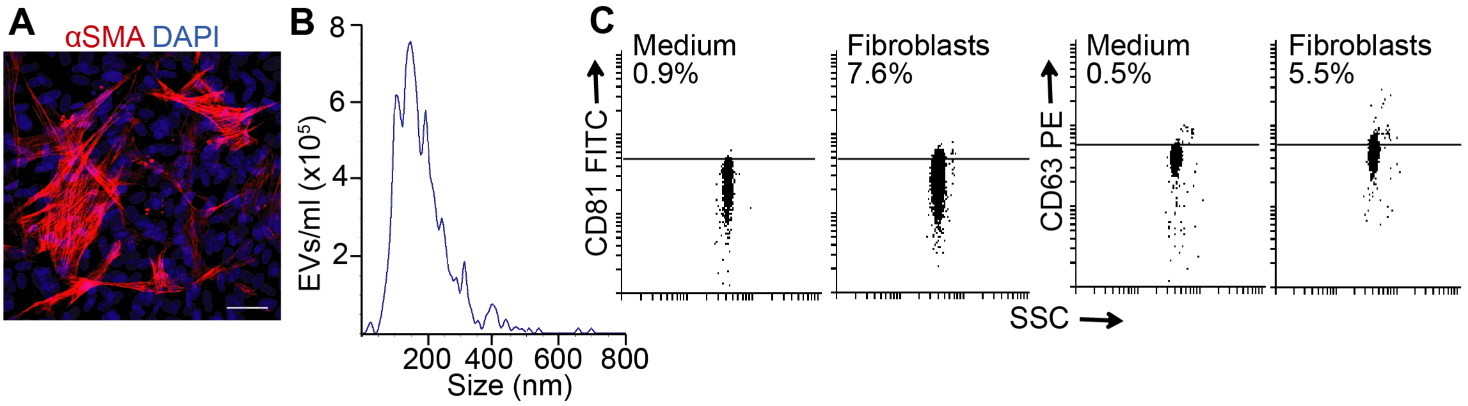

Figure S6. LUAD fibroblasts produce EVs. A) αSMA immunostaining of LUAD fibroblasts. B) NTA analysis of the conditioned medium from a LUAD fibroblast culture. The supernatant was measured after centrifugation at 12,500g. C) Percentage of anti-CD81 and anti-CD63-coated beads after incubating them in medium or in the supernatant of LUAD fibroblast cultures, detected with flow cytometry. The numbers indicate the percentage of positive beads. Scale bar: 50µm (A).

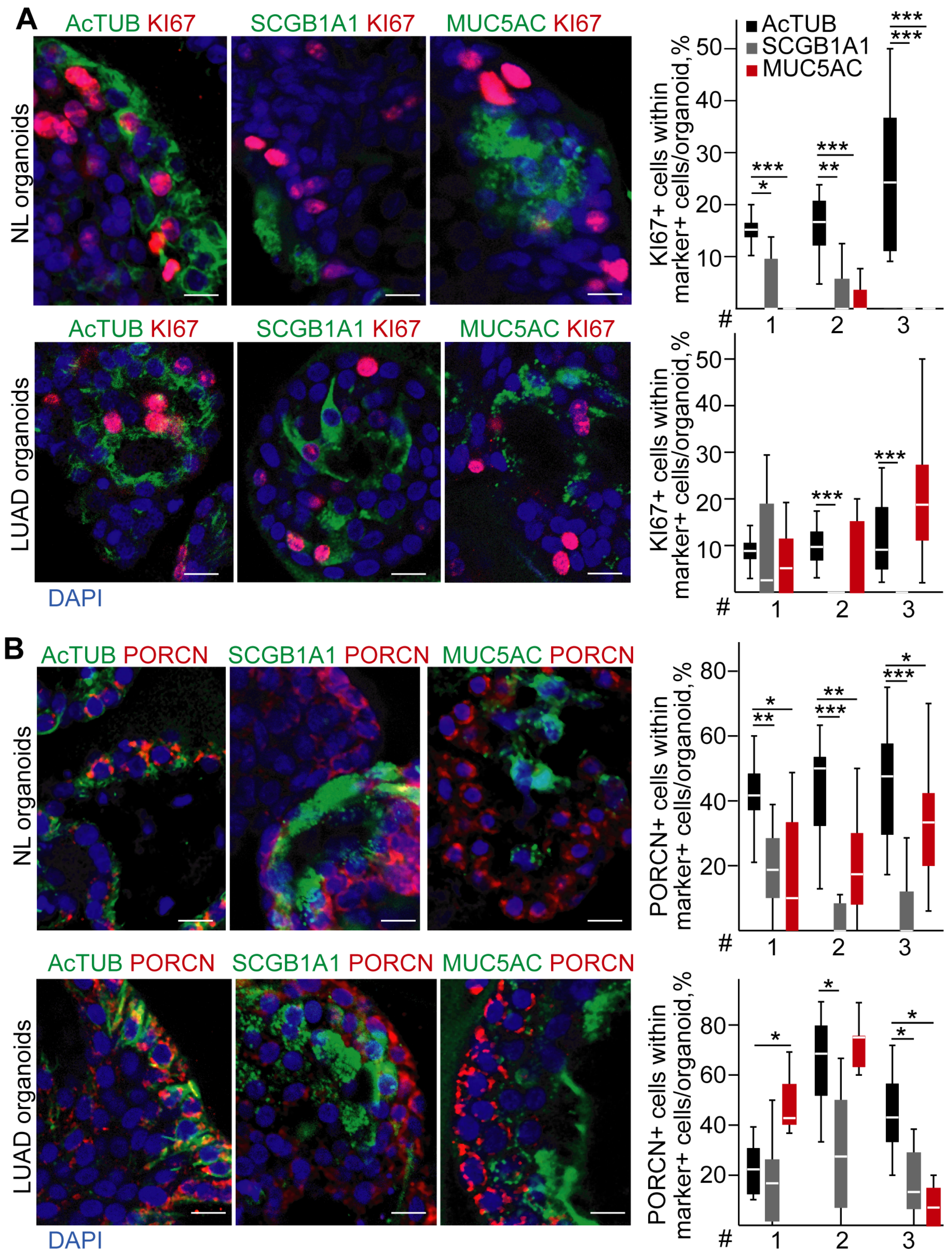

Figure S7. The distribution of proliferating and PORCN+ normal human bronchiolar (NL) or LUAD organoid cells within cell populations characterized with cell type markers. A-B) Representative confocal microscopic images and their evaluation from normal or LUAD organoids (n=10-12 from two replicates). Cell type specific markers were co-immunostained with KI67 (A) or PORCN (B). Note that when quantifying the images, KI67 (A) or PORCN+ (B) cells were counted within the cell populations indicated by different markers for each organoid. Sample# shows different patient-derived organoid lines. Kruskal-Wallis and Dunn' tests were used with *p<0.05, **p<0.01 and ***p<0.005. Scale bars: 20μm.

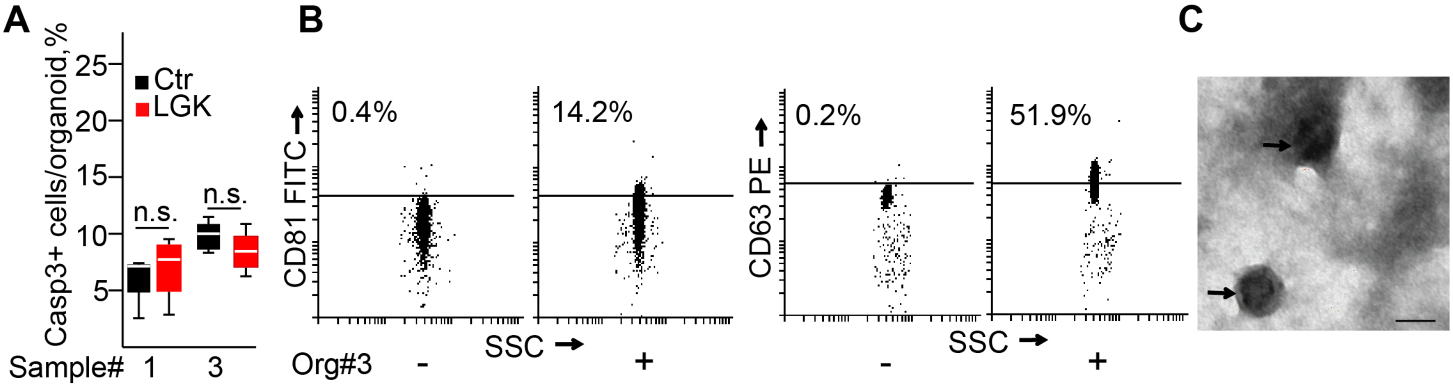

Figure S8. LUAD organoids produce EVs. A) The percentage of active caspase-3+ cells/organoid in two patient-derived LUAD organoid lines with/without LGK974 treatment (LGK) (quantification of confocal images, n=8-10 from two replicates). B) Anti-CD81 or anti-CD63-coated beads were incubated in medium with (+) or without (-) organoids (sample#3). C) Transmission electron microscopy (TEM) of the untracentrifuged pellet from organoid conditioned medium. The arrows show EVs. Mann-Whitney U-test with n.s.: $p>0.05$ was used (A). Scale bar: 100nm (C).
